# 1,1′-Dimethyl-4,4′-bipyridinium bis­(triiodide)

**DOI:** 10.1107/S1600536809015207

**Published:** 2009-04-30

**Authors:** Tuoping Hu

**Affiliations:** a Department of Chemistry, North University of China, Taiyuan, Shanxi 030051, People’s Republic of China

## Abstract

In the title compound, C_12_H_14_N_2_^2+^·2I_3_^−^, the 1,1′-dimethyl-4,4′-bipyridinium (DMBP) dication is charge balanced by two triiodide ions. The DMBP dication is planar within 0.010 (5) Å. The asymmetric unit contains only half of the dication, the other half being generated by an inversion center. Weak C—H⋯I inter­actions link the ions into sheets parallel to (121).

## Related literature

For a dication with similar geometry, see: Russell & Wallwork (1972 ▶). For anions with comparable geometry, see: Marsh (2004 ▶); Madsen *et al.* (1999 ▶).
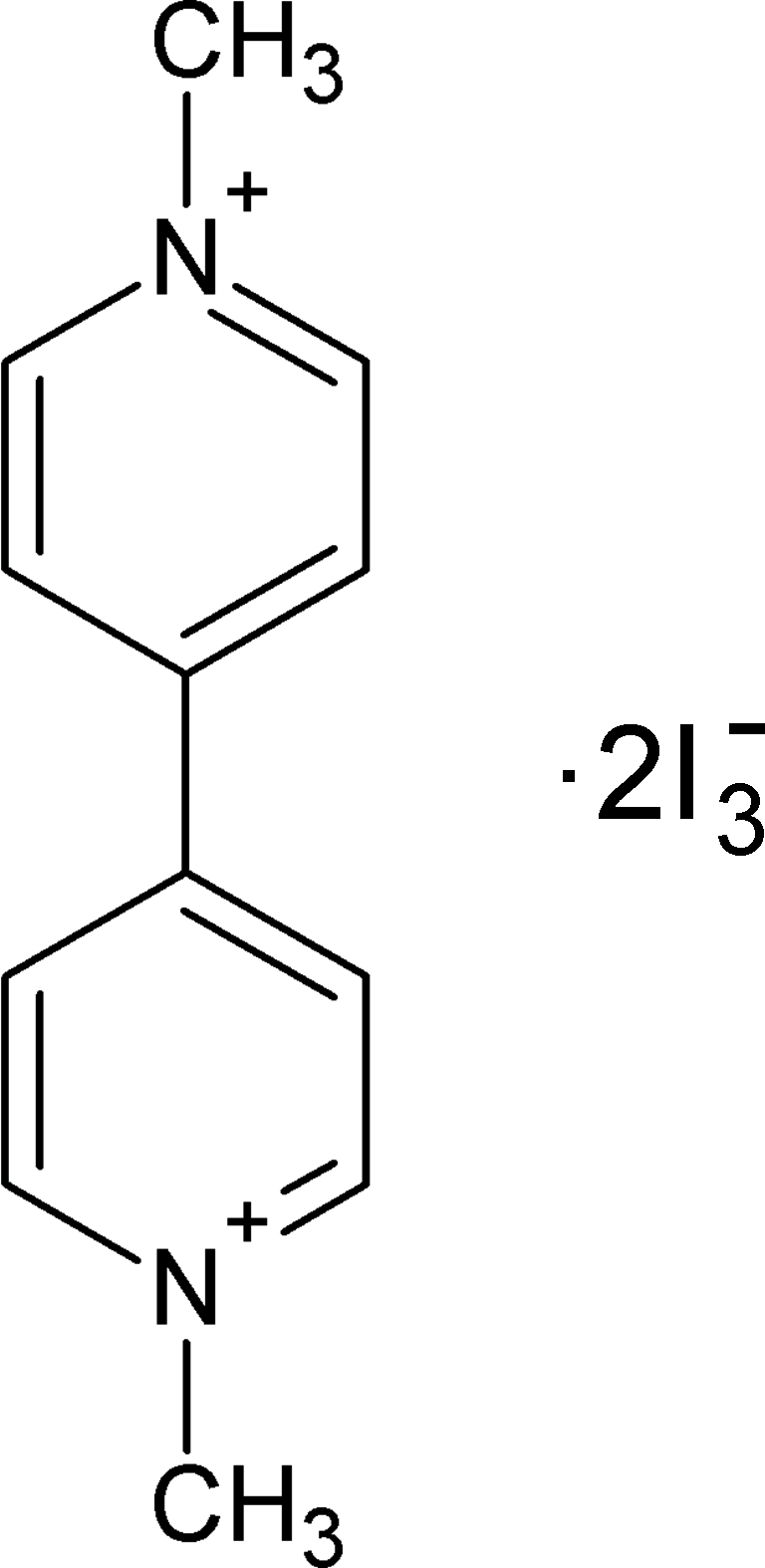


## Experimental

### 

#### Crystal data

C_12_H_14_N_2_^2+^·2I_3_^−^
*M* *_r_* = 947.65
Triclinic, 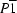

*a* = 7.5457 (4) Å
*b* = 7.9541 (6) Å
*c* = 9.3029 (6) Å
α = 90.306 (5)°
β = 94.192 (4)°
γ = 102.332 (5)°
*V* = 543.88 (6) Å^3^
*Z* = 1
Mo *K*α radiation
μ = 8.56 mm^−1^
*T* = 296 K
0.22 × 0.16 × 0.08 mm

#### Data collection

Bruker SMART CCD area-detector diffractometer
Absorption correction: multi-scan (*SADABS*; Sheldrick, 1996 ▶) *T*_min_ = 0.211, *T*_max_ = 0.504
12956 measured reflections
2683 independent reflections
1468 reflections with *I* > 2σ(*I*)
*R*_int_ = 0.052

#### Refinement

*R*[*F* ^2^ > 2σ(*F* ^2^)] = 0.040
*wR*(*F* ^2^) = 0.073
*S* = 1.02
2683 reflections
93 parameters
H-atom parameters constrained
Δρ_max_ = 0.97 e Å^−3^
Δρ_min_ = −0.86 e Å^−3^

### 

Data collection: *SMART* (Bruker, 2007 ▶); cell refinement: *SAINT-Plus* (Bruker, 2007 ▶); data reduction: *SAINT-Plus*; program(s) used to solve structure: *SHELXS97* (Sheldrick, 2008 ▶); program(s) used to refine structure: *SHELXL97* (Sheldrick, 2008 ▶); molecular graphics: *SHELXTL* (Sheldrick, 2008 ▶); software used to prepare material for publication: *SHELXTL*.

## Supplementary Material

Crystal structure: contains datablocks global, I. DOI: 10.1107/S1600536809015207/ez2167sup1.cif

Structure factors: contains datablocks I. DOI: 10.1107/S1600536809015207/ez2167Isup2.hkl

Additional supplementary materials:  crystallographic information; 3D view; checkCIF report

## Figures and Tables

**Table:** 

I1—I2	2.9341 (8)
I2—I3	2.9061 (8)

**Table:** 

I3—I2—I1	177.49 (2)

**Table 2:** Hydrogen-bond geometry (Å, °)

*D*—H⋯*A*	*D*—H	H⋯*A*	*D*⋯*A*	*D*—H⋯*A*
C3—H3⋯I3^i^	0.93	3.05	3.951 (8)	163
C2—H2⋯I1^ii^	0.93	3.16	4.066 (8)	164
C5—H5⋯I2^i^	0.93	3.13	3.839 (7)	135

Symmetry codes: (i) 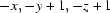; (ii) 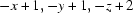.

**Fig. 1.**
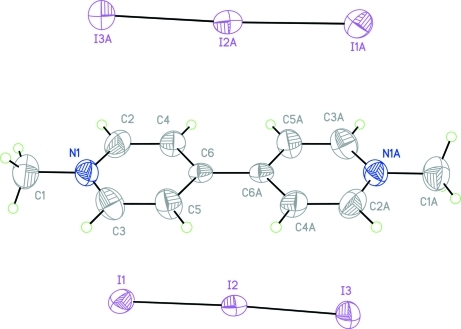
Molecular structure showing 50% probability displacement ellipsoids. The atoms marked with A are derived from the reference atoms by means of the (1 - x, 1 - y, 1 - z) symmetry transformation..

**Fig. 2.**
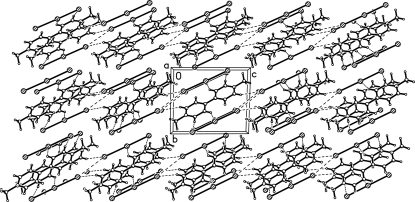
Packing diagram viewed down the a axis. Weak C—H···I interactions are shown as dotted lines.

## supplementary crystallographic information

### Comment

The title compound, (I), was obtained by chance when we tried to prepare the salt of the Pb(II) cation and DMBP in MeOH. This paper provides the first crystal structure of the DMBP dication with two triiodide anions.

Only half of the dication of DMBP is contained in the asymmetric unit, while the other half is generated by the inversion center at (1/2,1/2,1/2) (Fig 1.). The *N*,*N*'-dimethyl-4,4'bipyridylium(II) dication has an essentially planar conformation, the maximum deviation of the C1 atom (the methyl group) from its mean plane being 0.010 (5) Å. The geometry of the dication is similar to the one observed in Russell & Wallwork (1972). Meanwhile, the geometry of the anion is comparable to that described in Marsh (2004) and Madsen *et al.* (1999).

Weak C3—H3···I3 interactions link two I_3_ anions to each dication. A weaker C2—I2···H1 interaction links each anion to a further DMBP cation, to form sheets parallel to (121). Adjacent sheets are packed into a three-dimensional motif (Fig. 2).

### Experimental

C_12_H_14_N_2_.4Cl (0.5 mmol, 128 mg) and KI (10 mmol, 1660 mg) were added to 50 ml of CH_3_CN. After stirring and refluxing for 12 h, the mixture was filtered, and the clear solution was allowed to evaporate slowly under inert atmosphere. Prismatic crystals of the title compound were obtained after 5 days. The crystals were filtered, washed by cool EtOH and dried in air.

### Refinement

All of the H atoms were positioned geometrically and refined using a riding model with C—H = 0.930 Å and 0.96 Å, with *U*_iso_(H) = 1.2 and 1.5 times *U*_eq_(C), for aromatic and methyl hydrogens, respectively.

### Crystal data

**Table:**

C_12_H_14_N_2_^2+^·2I_3_^−^	*Z* = 1
*M**_r_* = 947.65	*F*(000) = 418
Triclinic, *P*1	*D*_x_ = 2.893 Mg m^−^^3^
Hall symbol: -P 1	Mo *K*α radiation, λ = 0.71073 Å
*a* = 7.5457 (4) Å	Cell parameters from 4412 reflections
*b* = 7.9541 (6) Å	θ = 2.6–27.6°
*c* = 9.3029 (6) Å	µ = 8.56 mm^−^^1^
α = 90.306 (5)°	*T* = 296 K
β = 94.192 (4)°	Prism, black
γ = 102.332 (5)°	0.22 × 0.16 × 0.08 mm
*V* = 543.88 (6) Å^3^	

### Data collection

**Table:**

Bruker SMART CCD area-detector diffractometer	2683 independent reflections
Radiation source: fine-focus sealed tube	1468 reflections with *I* > 2σ(*I*)
graphite	*R*_int_ = 0.052
φ and ω scans	θ_max_ = 28.3°, θ_min_ = 3.9°
Absorption correction: multi-scan (*SADABS*; Sheldrick, 1996)	*h* = −10→10
*T*_min_ = 0.211, *T*_max_ = 0.504	*k* = −10→10
12956 measured reflections	*l* = −11→12

### Refinement

**Table:**

Refinement on *F*^2^	Secondary atom site location: difference Fourier map
Least-squares matrix: full	Hydrogen site location: inferred from neighbouring sites
*R*[*F*^2^ > 2σ(*F*^2^)] = 0.040	H-atom parameters constrained
*wR*(*F*^2^) = 0.073	*w* = 1/[σ^2^(*F*_o_^2^) + (0.005*P*)^2^ + 2.2853*P*] where *P* = (*F*_o_^2^ + 2*F*_c_^2^)/3
*S* = 1.02	(Δ/σ)_max_ < 0.001
2683 reflections	Δρ_max_ = 0.97 e Å^−^^3^
93 parameters	Δρ_min_ = −0.86 e Å^−^^3^
0 restraints	Extinction correction: *SHELXL97* (Sheldrick, 2008), Fc^*^=kFc[1+0.001xFc^2^λ^3^/sin(2θ)]^-1/4^
Primary atom site location: structure-invariant direct methods	Extinction coefficient: 0.0028 (3)

### Special details

**Table:**

Geometry. All e.s.d.'s (except the e.s.d. in the dihedral angle between two l.s. planes) are estimated using the full covariance matrix. The cell e.s.d.'s are taken into account individually in the estimation of e.s.d.'s in distances, angles and torsion angles; correlations between e.s.d.'s in cell parameters are only used when they are defined by crystal symmetry. An approximate (isotropic) treatment of cell e.s.d.'s is used for estimating e.s.d.'s involving l.s. planes.
Refinement. Refinement of *F*^2^ against ALL reflections. The weighted *R*-factor *wR* and goodness of fit *S* are based on *F*^2^, conventional *R*-factors *R* are based on *F*, with *F* set to zero for negative *F*^2^. The threshold expression of *F*^2^ > σ(*F*^2^) is used only for calculating *R*-factors(gt) *etc*. and is not relevant to the choice of reflections for refinement. *R*-factors based on *F*^2^ are statistically about twice as large as those based on *F*, and *R*- factors based on ALL data will be even larger.

### Fractional atomic coordinates and isotropic or equivalent isotropic displacement parameters (Å^2^)

**Table:**

	*x*	*y*	*z*	*U*_iso_*/*U*_eq_	
I1	0.11371 (7)	0.64766 (7)	0.84204 (6)	0.0705 (2)	
I2	0.19121 (6)	0.80427 (6)	0.56237 (6)	0.05927 (17)	
I3	0.25337 (8)	0.96496 (8)	0.28546 (6)	0.0816 (2)	
N1	0.3773 (9)	0.2800 (7)	0.8128 (7)	0.0588 (16)	
C1	0.3276 (13)	0.1852 (11)	0.9438 (9)	0.085 (3)	
H1A	0.4248	0.2166	1.0179	0.128*	
H1B	0.3064	0.0638	0.9235	0.128*	
H1C	0.2190	0.2131	0.9758	0.128*	
C2	0.5358 (12)	0.3875 (11)	0.8116 (9)	0.074 (2)	
H2	0.6149	0.4034	0.8944	0.088*	
C3	0.2652 (11)	0.2566 (10)	0.6956 (10)	0.072 (2)	
H3	0.1526	0.1813	0.6969	0.086*	
C4	0.5864 (9)	0.4764 (10)	0.6903 (8)	0.061 (2)	
H4	0.6984	0.5532	0.6924	0.074*	
C5	0.3120 (10)	0.3414 (10)	0.5722 (8)	0.066 (2)	
H5	0.2309	0.3216	0.4906	0.079*	
C6	0.4743 (8)	0.4540 (7)	0.5658 (7)	0.0396 (14)	

### Atomic displacement parameters (Å^2^)

**Table:**

	*U*^11^	*U*^22^	*U*^33^	*U*^12^	*U*^13^	*U*^23^
I1	0.0689 (4)	0.0899 (4)	0.0576 (3)	0.0291 (3)	0.0010 (3)	0.0076 (3)
I2	0.0503 (3)	0.0648 (3)	0.0672 (3)	0.0224 (2)	0.0040 (2)	0.0045 (2)
I3	0.0838 (4)	0.0895 (4)	0.0816 (4)	0.0335 (3)	0.0267 (3)	0.0292 (3)
N1	0.066 (4)	0.051 (4)	0.063 (4)	0.016 (3)	0.016 (4)	0.010 (3)
C1	0.106 (7)	0.076 (6)	0.075 (6)	0.017 (5)	0.017 (5)	0.015 (5)
C2	0.071 (6)	0.088 (6)	0.061 (5)	0.020 (5)	−0.011 (4)	0.015 (5)
C3	0.062 (5)	0.066 (5)	0.078 (6)	−0.010 (4)	0.011 (5)	−0.003 (5)
C4	0.039 (4)	0.077 (5)	0.058 (5)	−0.004 (4)	−0.016 (3)	0.006 (4)
C5	0.052 (5)	0.080 (6)	0.054 (5)	−0.007 (4)	−0.003 (4)	−0.001 (4)
C6	0.031 (3)	0.032 (3)	0.054 (4)	0.005 (3)	−0.002 (3)	−0.003 (3)

### Geometric parameters (Å, °)

**Table:**

I1—I2	2.9341 (8)	C2—H2	0.9300
I2—I3	2.9061 (8)	C3—C5	1.364 (10)
N1—C2	1.314 (9)	C3—H3	0.9300
N1—C3	1.317 (9)	C4—C6	1.371 (8)
N1—C1	1.467 (9)	C4—H4	0.9300
C1—H1A	0.9600	C5—C6	1.359 (9)
C1—H1B	0.9600	C5—H5	0.9300
C1—H1C	0.9600	C6—C6^i^	1.464 (12)
C2—C4	1.370 (10)		
			
I3—I2—I1	177.49 (2)	N1—C3—C5	120.9 (7)
C2—N1—C3	119.7 (7)	N1—C3—H3	119.5
C2—N1—C1	119.8 (7)	C5—C3—H3	119.5
C3—N1—C1	120.5 (7)	C2—C4—C6	121.0 (6)
N1—C1—H1A	109.5	C2—C4—H4	119.5
N1—C1—H1B	109.5	C6—C4—H4	119.5
H1A—C1—H1B	109.5	C6—C5—C3	121.6 (7)
N1—C1—H1C	109.5	C6—C5—H5	119.2
H1A—C1—H1C	109.5	C3—C5—H5	119.2
H1B—C1—H1C	109.5	C5—C6—C4	115.9 (6)
N1—C2—C4	121.0 (7)	C5—C6—C6^i^	122.1 (7)
N1—C2—H2	119.5	C4—C6—C6^i^	122.1 (7)
C4—C2—H2	119.5		
			
C3—N1—C2—C4	0.3 (12)	N1—C3—C5—C6	−0.7 (13)
C1—N1—C2—C4	179.4 (7)	C3—C5—C6—C4	0.0 (11)
C2—N1—C3—C5	0.6 (12)	C3—C5—C6—C6^i^	−179.8 (8)
C1—N1—C3—C5	−178.5 (7)	C2—C4—C6—C5	0.9 (11)
N1—C2—C4—C6	−1.0 (12)	C2—C4—C6—C6^i^	−179.3 (8)

Symmetry codes: (i) −*x*+1, −*y*+1, −*z*+1.

### Hydrogen-bond geometry (Å, °)

**Table:**

*D*—H···*A*	*D*—H	H···*A*	*D*···*A*	*D*—H···*A*
C3—H3···I3^ii^	0.93	3.05	3.951 (8)	163
C2—H2···I1^iii^	0.93	3.16	4.066 (8)	164
C5—H5···I2^ii^	0.93	3.13	3.839 (7)	135

Symmetry codes: (ii) −*x*, −*y*+1, −*z*+1; (iii) −*x*+1, −*y*+1, −*z*+2.

## References

[bb1] Bruker (2007). *SMART* and *SAINT-Plus* Bruker AXS Inc., Madison, Wisconsin, USA.

[bb2] Madsen, D., Burghammer, M., Fiedler, S. & Müller, H. (1999). *Acta Cryst.* B**55**, 601–606. 10.1107/s0108768199003468 10927401

[bb3] Marsh, R. E. (2004). *Acta Cryst.* B**60**, 252–253. 10.1107/S0108768104003878 15017100

[bb4] Russell, J. H. & Wallwork, S. C. (1972). *Acta Cryst.* B**28**, 1527–1533.

[bb5] Sheldrick, G. M. (1996). *SADABS* University of Göttingen, Germany.

[bb6] Sheldrick, G. M. (2008). *Acta Cryst.* A**64**, 112–122. 10.1107/S0108767307043930 18156677

